# Low molecular weight heparin versus unfractioned heparin for anticoagulation during perioperative extracorporeal membrane oxygenation: A single center experience in 102 lung transplant patients

**DOI:** 10.1111/aor.13642

**Published:** 2020-02-18

**Authors:** Johannes Gratz, André Pausch, Eva Schaden, Andreas Baierl, Peter Jaksch, Friedrich Erhart, Konrad Hoetzenecker, Marion Wiegele

**Affiliations:** ^1^ Department of Anesthesiology, Intensive Care and Pain Medicine Medical University of Vienna Vienna Austria; ^2^ Department of Statistics and Operations Research University of Vienna Vienna Austria; ^3^ Department of Thoracic Surgery Medical University of Vienna Vienna Austria; ^4^ Department of Neurosurgery Medical University of Vienna Vienna Austria

**Keywords:** alternative anticoagulants, anticoagulation, extracorporeal membrane oxygenation, low molecular weight heparin, lung transplantation, unfractioned heparin

## Abstract

Extracorporeal membrane oxygenation (ECMO) is gaining importance in the perioperative management of lung transplant patients. To date, the ideal substance for anticoagulation of ECMO patients is still a matter of debate. In this study, we describe our experience with the use of low molecular weight heparin (LMWH) in comparison with unfractioned heparin (UFH) in lung transplant patients undergoing perioperative ECMO support. We retrospectively analyzed data from all lung transplant patients who underwent perioperative ECMO support at our institution between 2013 and 2017. Bleeding events served as primary outcome parameter. Secondary outcome parameters consisted of thromboembolic events. 102 patients were included in this study, of which 22 (21.6%) received UFH for anticoagulation, and 80 (78.4%) received LMWH. There was no difference between the two groups in regard to serious bleeding events (22.7% in the UFH group vs 12.5% in the LMWH group, *P = *.31). However, the proportion of patients experiencing thromboembolic events was significantly higher in the UFH group than in the LMWH group (50% vs 20%, *P = *.01). After adjusting for baseline differences between the two groups, we still observed a difference with respect to thromboembolic events. These data remain to be validated in future prospective, randomized trials.

## 1 INTRODUCTION

Lung transplantation is the only potentially life‐saving therapy in a selected number of patients with typical end‐stage lung diseases.^1^, ^2^ Extracorporeal membrane oxygenation (ECMO) is gaining importance in the intra‐ and perioperative management of lung transplant patients, for example, as preoperative bridge‐to‐transplant or postoperative bridge‐to‐recovery in case of primary organ malfunction or prophylactic prolongation in high‐risk patients.^3^, ^4^, ^5^ Common complications of ECMO therapy include bleeding events as well as thromboembolic events.^6^ A number of factors play a role in disturbing the fragile balance between bleeding and thrombosis during ECMO support and can thus lead to hemorrhagic as well as thromboembolic complications.^7^, ^8^ To date, the ideal strategy for anticoagulation during ECMO therapy remains unclear; however, the Extracorporeal Life Support Organization (ELSO) guideline on anticoagulation primarily suggests the use of unfractioned heparin (UFH).^9^, ^10^, ^11^

In intensive care medicine, low molecular weight heparins (LMWH) have replaced UFH in most indications due to a number of beneficial characteristics, such as predictable pharmacokinetics and no need for routine drug monitoring.^12^, ^13^ Safety and efficacy of LMWH for anticoagulation of extracorporeal circuits have been shown in the setting of renal replacement therapy.^14^, ^15^ Data on the use of LMWH for anticoagulation during ECMO remain scarce with a single study evaluating LMWH in prophylactic dosage for anticoagulation of 60 nonsurgical ECMO patients.^16^

At our institution, LMWH has been the substance of first choice for anticoagulation of ECMO patients since 2010. As per local protocol, it is given in a half‐therapeutic dose regimen split into 2 × 0.5 mg enoxaparin/kg bodyweight/day. However, UFH is still being used at the discretion of the attending intensive care physician. Thus, the aim of our study was to retrospectively compare the risk of hemorrhagic and thromboembolic events in lung transplant patients undergoing perioperative ECMO anticoagulated with LMWH versus those anticoagulated by means of UFH.

## 2 PATIENTS AND METHODS

This investigator‐initiated, retrospective, observational cohort study was conducted at the Medical University of Vienna, Austria, a tertiary care center. The study was performed in accordance with the Declaration of Helsinki, approved by the ethics committee of the Medical University of Vienna (EK1017/2017) and registered at the German Clinical Trials Register (DRKS00013593). The design and conduct of the study followed the STROBE checklist for cohort studies.

### 2.1 Procedures

Patients included in this study underwent one of four types of perioperative ECMO support:

- preoperative ECMO (ie, the ECMO was implanted preoperatively at the intensive care unit [ICU] as bridge‐to‐transplant and successfully discontinued in the operating room at the end of lung transplantation.)
- pre‐ and postoperative ECMO (ie, the ECMO was implanted preoperatively at the ICU as bridge‐to‐transplant and the patient was readmitted to the ICU postoperatively with ongoing ECMO support.)
- prolonged ECMO (ie, the ECMO was implanted intraoperatively and the patient was admitted to the ICU postoperatively with ongoing ECMO support.)
- postoperative ECMO (ie, patient requiring de novo ECMO implantation in the early postoperative period.)

The decision for ECMO support was made on an individual basis by attending intensive care physicians and thoracic surgeons. According to the indication of ECMO therapy, a venoarterial or venovenous system was implanted either surgically or percutaneously. A heparin‐coated system was used in all cases. During implantation of ECMO, all patients received a bolus of UFH. Subsequently, anticoagulation with subcutaneous enoxaparin or intravenous UFH was started at the intensivist's discretion in consultation with the thoracic surgeons according to the patients' individual risk of bleeding. Generally, anticoagulation was started either immediately (preoperative ECMO) or within 24 hours after surgery, given there was adequate hemostasis (prolonged or postoperative ECMO). As per local protocol, LMWH was given in a fixed half therapeutic dose regimen of 2 × 0.5 mg enoxaparin/kg bodyweight/day without guidance by antiXa values. Dosing of UFH was guided by at least twice daily measurements of activated partial thromboplastin time (APTT) aiming for goal values of 1.5 times the baseline value.

### 2.2 Data collection

All patients who underwent lung transplantation at our institution between 2013 and 2017 were screened for the use of perioperative ECMO. According to the anticoagulation used, patients who underwent perioperative ECMO support were divided into two groups: those anticoagulated by means of subcutaneous administration of LMWH and those anticoagulated by UFH administered intravenously.

Baseline demographic and clinical data were recorded on the day when anticoagulation for ECMO support was started with either subcutaneous LMWH or continuous intravenous UFH. Patients were followed and relevant parameters recorded daily until (a) ECMO was discontinued, (b) anticoagulation was switched to a different substance, or (c) anticoagulation was withheld for ≥1 day.

### 2.3 Outcome parameters and statistical analyses

The incidence of serious bleeding events served as *primary outcome parameter*. Serious bleeding events were defined as

- bleeding that required any form of surgical intervention,
- intracranial bleeding,
- uncontrollable bleeding that led to death.

Thromboembolic events and a composite outcome of hemorrhagic together with thromboembolic events served as *secondary outcome parameters*. Thromboembolic events were defined as

- arterial thromboembolic events, including myocardial infarction and cerebral stroke,
- venous thromboembolic events, including deep vein thrombosis and pulmonary embolism,
- circuit‐related thrombosis (including oxygenator exchanges).

After completion of data collection, we decided to conduct post hoc analyses of packed red blood cells (PRBCs)‐requirements. Because of significantly different ECMO durations and timings (ie, pre‐ vs postoperative ECMO therapy) in the two groups, post hoc time‐specific analyses of recorded variables were carried out for the first five days of the study period in both groups and outcomes corrected for timing of ECMO therapy by logistic regression. All post hoc analyses are marked as such in the results section.

Distributions of all metric variables were assessed visually by quantile‐quantile plots. Normally distributed variables are described as mean ± standard deviation (SD), whereas non‐normally distributed metric variables are summarized by median and interquartile range (IQR). *χ* ^2^‐tests were used to test for differences between anticoagulation treatment and categorical variables while unpaired *t*‐test or Mann‐Whitney tests, respectively, were applied to metric variables according to data distributions. Logistic regression models were estimated to assess effects of anticoagulation treatment adjusted for ECMO timing. All tests were two‐sided and *P* values less than .05 were considered statistically significant. Statistical analyses were performed with the statistical software R version 3.50 (R Development Core Team, 2018), and GraphPad Prism Version 6.0 (GraphPad Software, Inc, LaJolla, CA, USA).

## 3 RESULTS

**Figure 1:**
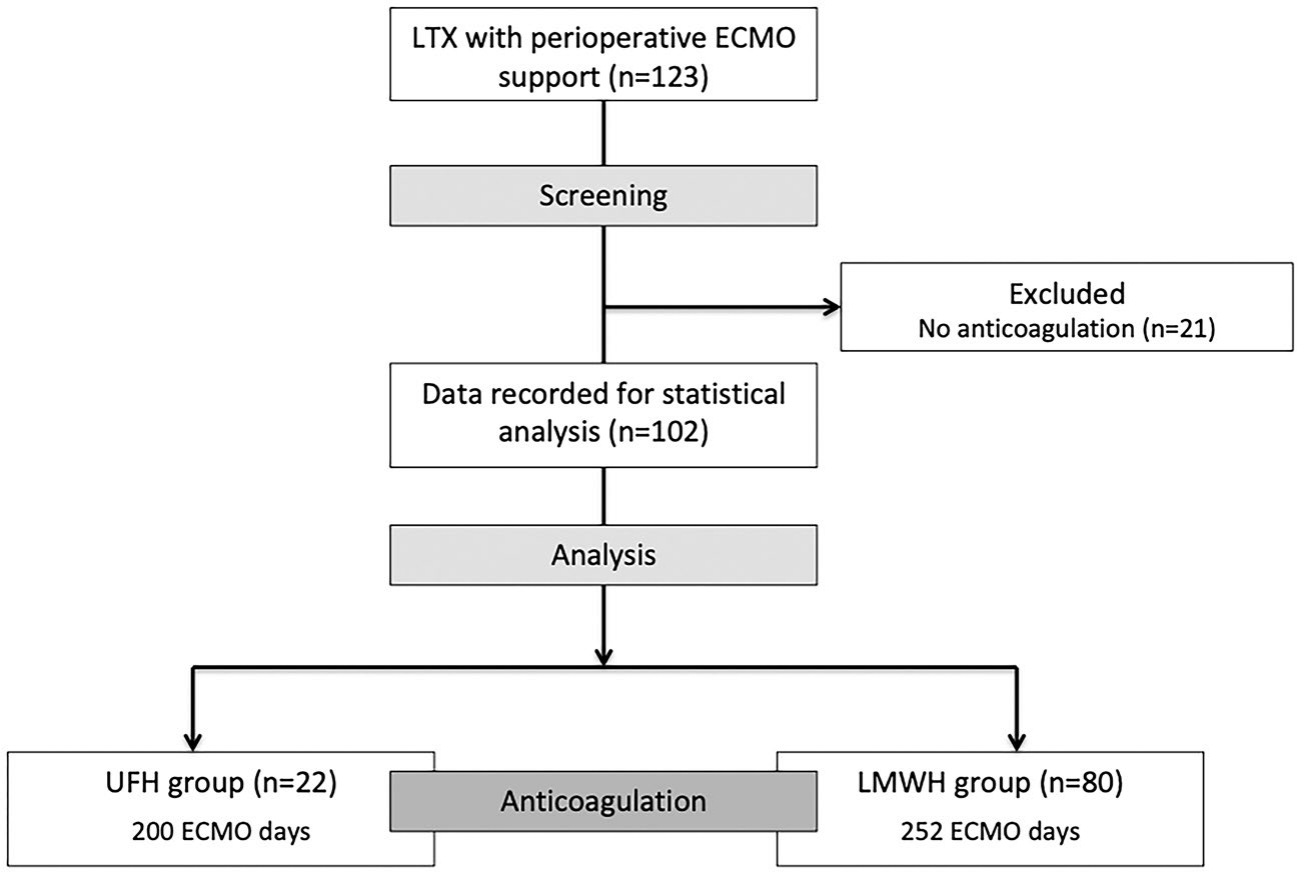
Inclusion and dropout of patients as well as type of anticoagulant used. ECMO, extracorporeal membrane oxygenation; LMWH, low molecular weight heparin; LTX, lung transplantation; UFH, unfractioned heparin

In this study, 123 lung transplant patients who underwent perioperative ECMO support between 2013 and 2017 were screened for inclusion. Figure 1 shows a flowchart depicting the inclusion and dropout of patients, as well as the type of anticoagulation. All patients in the UFH group received continuous infusions of UFH throughout the study period.

Table 1 shows baseline and demographic characteristics of the patients included. The two groups were similar regarding sex, age, weight, preexisting coagulation disorders, indication for lung transplantation, as well as ECMO and ICU mortality. However, the median length of ICU stay was longer in the UFH group. Patients in the UFH group had a longer total duration of ECMO therapy, as well as a longer preoperative period of ECMO support. Importantly, the study period ‐ defined by ECMO days with first anticoagulant used ‐ was also longer in the UFH group.

**Table 1:** Baseline and demographic characteristics of patients included

Characteristics	UFH (n = 22)	LMWH (n = 80)	*P*
Male sex	54.5%	53.8%	1.00
Age (years)	41 ± 14	40 ± 15	.72
Weight (kg)	64 ± 17	61 ± 17	.46
BMI (kg m^−2^)	22 ± 4	22 ± 4	.97
Preexisting coagulation disorder	9.1%	5.0%	.61
Indication for LTX			.87
CF	22.7%	23.7%	
Fibrosis	36.4%	26.3%	
PPH	22.7%	28.8%	
COPD	4.5%	8.7%	
Other	13.7%	12.5%	
Length of ICU stay (days)	36 (30)	24 (19)	**<.01**
ICU mortality	22.7%	8.8%	.13
ECMO mortality	9.1%	2.5%	.20
ECMO days			
Total	7 (12)	4 (3)	**<.01**
Preoperative	7 (7)	0 (1)	**<.01**
Postoperative	2 (5)	3 (2)	.21
Timing of ECMO support			**<.01**
Preoperative	36.4%	15%	
Pre‐ and postoperative	45.5%	11.3%	
Prolonged	13.6%	70%	
Postoperative	4.5%	3.7%	
ECMO days before first anticoagulant	1 (2)	1 (1)	.27
ECMO days with first anticoagulant	6 (4)	2 (2)	**<.01**

Note

Values are given as percentage, mean ± SD, or median (IQR) as appropriate. Significant *P* values have been marked in bold.

Abbreviations: BMI, body mass index; CF, cystic fibrosis; COPD, chronic obstructive pulmonary disease; ECMO, extracorporeal membrane oxygenation; ICU, intensive care unit; LTX, lung transplantation; PPH, primary pulmonary hypertension.

Table 2 shows the baseline parameters recorded on the first day patients received anticoagulation during ECMO (ie, the first day of the study period). Significantly more patients underwent venoarterial ECMO support in the LMWH group. Patients in the LMWH group had lower platelet, fibrinogen, and antithrombin levels, as well as higher creatinine and bilirubin levels. Patients in the LMWH group received a mean cumulative dose of 48 mg enoxaparin/day (0.8 mg/kg/24 hours), whereas patients in the UFH group received a mean cumulative dose of 15 586 IU UFH/day, equivalent to 649 IU/hour (10 IU/kg/hour). We observed no local complications, such as relevant hematomas or infections associated with the use of LMWH.

**Table 2:** Baseline characteristics on the first day of anticoagulation, that is, the first day of the study period

Characteristics	UFH (n = 22)	LMWH (n = 80)	*P*
VA ECMO	31.8%	80%	**<.01**
Hemoglobin (g/L)	109 ± 22	109 ± 16	.9
Platelets (10^9^/L)	196 (252)	94 (97)	**<.01**
PT (%)	65 ± 15	61 ± 19	.34
aPTT (s)	38 ± 9	41 ± 9	.16
TT (s)	16 (6)	15 (5)	.45
Fibrinogen (g/L)	5.0 (2.7)	2.8 (1.5)	**<.01**
AT (%)	81 ± 21	66 ± 19	**<.01**
Creatinine (nmol/L)	48 (20)	63 (38)	**.01**
Bilirubin (μmol/L)	11 (23)	31 (42)	**<.01**
Hemodynamics^a^	4 (1)	4 (1)	.20

Note

Values are given as percentage, mean ± SD, or median (IQR) as appropriate. Significant *P* values have been marked in bold.

Abbreviations: aPTT, activated partial thromboplastin time; AT, antithrombin; LMWH, low molecular weight heparin; PT, prothrombin time; SOFA, sequential organ failure assessment; TT, thrombin time; UFH, unfractioned heparin; VA ECMO, venoarterial extracorporeal membrane oxygenation.

a Points according to SOFA score.

### 3.1 Bleeding events

Fifteen of the 102 included patients (14.7%) experienced a total of 18 serious bleeding events, as defined in the Methods section. All events consisted in surgical interventions due to bleeding. No intracranial bleeding or uncontrollable fatal bleeding occurred during the study period. When comparing the incidence of serious bleeding events between the two groups the difference did not show statistical significance (22.7% in the UFH group vs 12.5% in the LMWH group, *P = *.31). The mean number of bleeding events per patient was 0.4 (±0.8) in the UFH group versus 0.1 (±0.3) in the LMWH group (*P = *.18). The mean number of bleeding events per patient per ECMO day was 0.03 (±0.09) in the UFH group versus 0.03 (±0.10) in the LMWH group (*P* = .32).

### 3.2 Thromboembolic events

A total of 42 thromboembolic events occurred in 27 of the 102 included patients (26.5%), of which the majority of 40 events consisted of circuit‐related thrombosis. While no myocardial infarction or deep vein thrombosis were observed, one ischemic cerebral stroke on day 7 of ECMO treatment and one pulmonary embolism on day 4 of ECMO treatment occurred in the UFH group. About 50% of the patients in the UFH group suffered from at least one thromboembolic event, whereas only 20% of the patients in the LMWH group experienced thromboembolic events (*P = *.01). The mean number of thromboembolic events per patient was 0.9 (±1.2) in the UFH group versus 0.3 (±0.6) in the LMWH group (*P = *.03). The mean number of thromboembolic events per patient per ECMO day was 0.11 (±0.16) in the UFH group versus 0.06 (±0.14) in the LMWH group (*P* = .01).

### 3.3 Composite outcome and post hoc analyses

The composite outcome of at least one bleeding or thromboembolic event occurred in a higher percentage of patients in the UFH group (59.1%), compared with the LMWH group (31.3%; *P = *.02). The mean number of both bleeding and thromboembolic events per patient per ECMO day was 0.14 (±0.19) in the UFH group versus 0.09 (±0.16) in the LMWH group (*P* = .06).

The post hoc analysis of transfusion requirements during the study period showed a mean administration of 0.84 PRBCs/24 hours/patient in the LMWH group, whereas in the UFH group 0.43 PRBCs/24 hours/patient were administered (*P *= .11).

Post hoc analyses of the combined outcome “serious bleeding event or >2 PRBCs/24 hours” showed no significant difference between the two groups (13.9% LMWH vs 9.6% UFH; *P = *.89). The total duration of ECMO therapy as well as the studied period of anticoagulation during ECMO support differed significantly between the two groups (Table 1). This was due to differences in timing of ECMO (Table 1), as preoperative ECMO therapy (ie, bridge‐to‐transplant) was associated with longer ECMO durations while waiting for a compatible organ. The runtime of ECMO therapy is a well described factor in the probability of complications, including thromboembolic events.^17^ Therefore, we decided to perform post hoc corrections for these relevant baseline differences between the two groups. First, we limited analysis to the first five days of the study period and second, we corrected for differences in timing of ECMO (eg, pre‐ vs postoperative) by means of logistic regression.

Table 3 shows relevant laboratory values and hemodynamic characteristics during days 2‐5 of anticoagulation compared between the two study groups. Apart from the aPTT, only creatinine levels showed significant differences throughout the period of five days.

**Table 3:** Laboratory values and hemodynamic characteristics during days 2‐5 of anticoagulation

Laboratory values	Day 2			Day 3			Day 4			Day 5		
	UFH (n = 22)	LMWH (n = 63)	*P*	UFH (n = 21)	LMWH (n = 41)	*P*	UFH (n = 16)	LMWH (n = 27)	*P*	UFH (n = 15)	LMWH (n = 17)	*P*
Hb	106 ± 17	102 ± 13	.31	104 ± 10	99 ± 14	.14	101 ± 11	97 ± 13	.25	95 ± 11	96 ± 12	.81
Plt	186 ± 98	96 ± 85	**<.01**	131 ± 72	80 ± 66	**<.01**	128 ± 62	85 ± 72	.05	118 ± 58	100 ± 68	.43
PT	62 ± 17	60 ± 16	.56	71 ± 20	88 ± 75	.19	70 ± 18	82 ± 19	.06	69 ± 18	80 ± 21	.1
**aPTT**	46 ± 11	41 ± 5	**.03**	50 ± 8	40 ± 7	**<.01**	59 ± 13	38 ± 5	**<.01**	55 ± 9	41 ± 14	**<.01**
TT	40 ± 36	17 ± 14	**.01**	36 ± 29	16 ± 3	**<.01**	44 ± 29	17 ± 4	**<.01**	36 ± 30	19 ± 5	.06
Fib	4.7 ± 1.7	3.5 ± 2.4	**.02**	4.5 ± 2.0	3.1 ± 1.4	**<.01**	4.3 ± 2.1	3.0 ± 1.7	.05	4.5 ± 1.8	3.0 ± 1.7	**.03**
AT	74 ± 18	67 ± 19	.15	73 ± 22	75 ± 18	.71	70 ± 19	84 ± 19	**.02**	75 ± 20	94 ± 20	**.01**
**Crea**	54 ± 31	77 ± 54	**<.01**	54 ± 84	85 ± 55	**<.01**	46 ± 23	84 ± 54	**<.01**	46 ± 23	85 ± 62	**.03**
Bili	27 ± 29	36 ± 34	.24	32 ± 42	29 ± 32	.84	25 ± 44	29 ± 39	.81	36 ± 63	34 ± 49	.98
Hemod^a^	3 ± 1	3 ± 1	.82	3 ± 1	3 ± 1	.28	3 ± 1	2 ± 1	.19	3 ± 1	3 ± 1	.28

Note

Values are given as mean ± SD. Significant *P* values have been marked in bold.

Abbreviations: aPTT, activated partial thromboplastin time (s); AT, antithrombin (%); Bili, bilirubin (μmol/L); Crea, creatinine (nmol/L); Fib, fibrinogen (g/L); Hb, hemoglobin (g/L); Hemod, hemodynamics; LMWH, low molecular weight heparin; Plt, platelets (10^9^/L); PT, prothrombin time (%); SOFA, sequential organ failure assessment; TT, thrombin time (s); UFH, unfractioned heparin.

a Points according to SOFA score.

**Figure 2:**
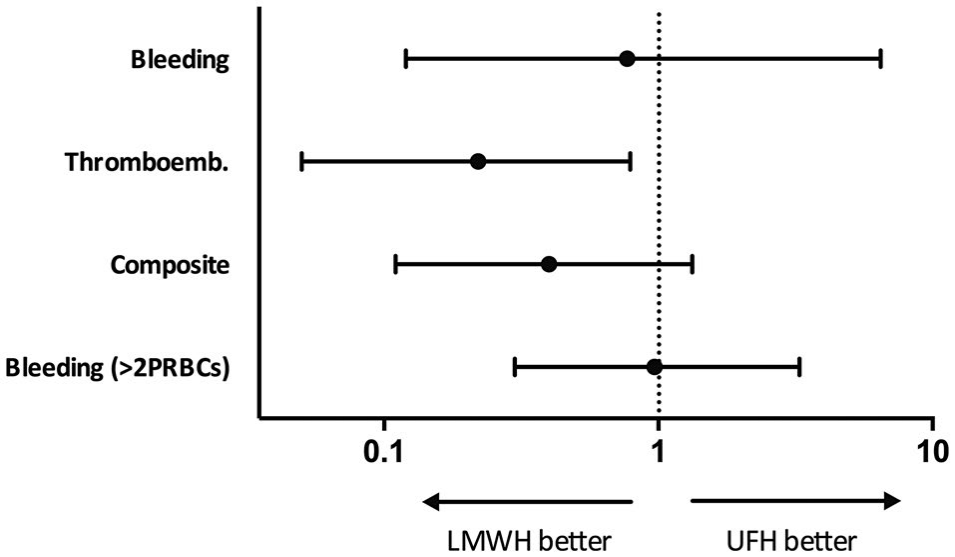
Effects of anticoagulation therapy on outcomes adjusted for ECMO timing and limited to the first five days of treatment. Odds ratios show effects of LMWH compared to UFH (95% CIs for ORs). LMWH, low molecular weight heparin; UFH, unfractioned heparin

Figure 2 shows the effects of anticoagulation therapy on the studied outcomes limited to the first five days of anticoagulation and adjusted for timing of ECMO by means of logistic regression. No difference occurred in regard to serious bleeding events (OR 0.77, 95% CI 0.12‐6.46; *P* = .79), also when including transfusion >2 PRBCs/24 hours (OR 0.97, 95% CI 0.30‐3.27; *P* = .96). Comparing thromboembolic events still showed a significant difference (OR 0.22, 95% CI 0.05‐0.79; *P* = .02) after correction for baseline discrepancies. However, after adjustment for ECMO duration and timing, we did not observe a significant difference in regard to the composite outcome (OR 0.40, 95% CI 0.11‐1.33; *P* = .14).

## 4 DISCUSSION

To the best of our knowledge, this is the first study to compare the use of LMWH with UFH for anticoagulation in perioperative ECMO support of lung transplant patients. We observed no differences in regard to risk of bleeding or transfusion requirements. However, patients who received LMWH subcutaneously had a lower risk of thromboembolic events compared to those anticoagulated by means of intravenous UFH.

These results are in line with large‐scale clinical trials and meta‐analyses in general ICU patients who showed reduced rates of thromboembolic events as well as an overall beneficial clinical effect of LMWH compared to UFH for thromboprophylaxis.^12^, ^13^, ^18^, ^19^ Safety and potential superiority of subcutaneously administered LMWH compared to intravenous UFH have also been described for therapeutic anticoagulation.^20^, ^21^ Additionally, safety and efficacy of LMWH have been demonstrated in the setting of anticoagulation for extracorporeal circuits, namely renal replacement therapy.^22^, ^23^ At our institution, LMWH has been the preferred substance for anticoagulation of ECMO patients for more than eight years. However, data on the use of LMWH for anticoagulation during ECMO therapy are scarce with one single‐center, observational study showing feasibility of prophylactic anticoagulation with enoxaparin during the course of venovenous ECMO in nonsurgical patients.^16^

Reasons for reluctance to use LMWH for anticoagulation in the setting of ECMO can only be subject of speculation. In the clinical context of our study, one reason might be the theoretical concern of its irreversibility compared to UFH in the immediate perioperative period. However, we observed no difference between groups with regard to serious bleeding events (LMWH 12.5% vs UFH 22.7%, *P = *.31). This mirrors the results of recent publications on the use of enoxaparin, including a Cochrane review on its perioperative use, showing a reduction of bleeding rates.^24^, ^25^ Reduced bleeding rates add to a number of other well described benefits with the use of LMWH, such as reduced rates of heparin‐induced thrombocytopenia, reduced activation of thrombocytes and improved bioavailability, ease of administration as well as reduced need for laboratory monitoring.^12^, ^13^, ^18^, ^19^, ^20^, ^21^, ^24^, ^25^

In our study, patients in the LMWH group received a mean dose of 0.8 mg/kg enoxaparin daily, which is closer to the recommended prophylactic dose than the dose suggested for therapeutic anticoagulation.^26^ ELSO guidelines suggest an initial dose of 7.5‐20 IU/kg/hour UFH for anticoagulation of ECMO patients.^11^ In our study, patients in the UFH group received a mean dose of 10 IU/kg/hour. APTT levels were significantly higher in the UFH group, ranging between 46 and 59 seconds compared to a baseline level of 38 seconds. Thus, patients in the LWMH group received lower than recommended dosages for therapeutic anticoagulation, whereas patients in the UFH group were within the intended range. Still, thromboembolic events occurred less frequently in the LMWH group.

Due to its retrospective nature, important limitations of this study need to be recognized. First of all, limited data quality did not allow for further analyses that would have been of interest. For example, while aPTT levels were recorded on a daily basis, we could not collect antiXa levels or ECMO flow rates in a reliable manner. Undeniably, flow rates play a relevant role in regard to the thromboembolic risk of ECMO patients. Second, relevant baseline differences occurred between the two groups. A significantly higher proportion of patients in the LMWH group (80%) were on venoarterial ECMO support, compared to the UFH group (31.8%, *P* < .01). In previous studies, venoarterial ECMO was associated with a higher risk of bleeding compared to a venovenous mode.^27^, ^28^, ^29^ Although one could, therefore, argue that patients in the LMWH group were at higher risk for bleeding, we observed no difference in bleeding between the two groups. Another discrepancy between the two groups arose from different ECMO timings: a higher proportion of patients in the UFH group underwent preoperative ECMO. This can be explained by a preference to use UFH in the preoperative bridge‐to‐transplant situation. Compared to preoperative patients, the postoperative phase is associated with a number of changes in the coagulation system due to surgery‐related factors, such as inflammatory response or the intraoperative use of procoagulant drugs.^30^ Furthermore, the different ECMO timings also led to significantly longer ECMO runtimes in the UFH group. The association between ECMO runtime and risk of thromboembolic as well as bleeding events has been described before.^16^, ^17^ However, after post hoc correction for the differences in ECMO timing and runtime, we still found a significantly lower risk of thromboembolic events in the LMWH group. Although we tried to correct for the differences by means of post hoc analyses, our results need to be viewed in the light of their retrospective nature and demand validation by future prospective, randomized trials. Despite its limitations, we still think that this study adds valuable information to the field of anticoagulation of ECMO patients.

## 5 CONCLUSION

In summary, this single center retrospective cohort study showed no difference in regard to bleeding risk between the use of LMWH and UFH for anticoagulation of perioperative ECMO patients. However, the use of LMWH was associated with a lower risk of thromboembolic events. Our data suggest that subcutaneous LMWH is a safe and viable alternative to intravenous UFH for anticoagulation during perioperative ECMO support in lung transplant patients.

## DISCLOSURE

JG has received support for congress travels and speaker fees from CSL Behring, Mitsubishi Tanabe Pharma and Johnson & Johnson Medical Products as well as advisory board honoraria from Instrumentation Laboratory Company. ES has received speaker fees from Baxalta Österreich GmbH, Boehringer Ingelheim RCV GmbH & Co KG Austria, Daiichi‐Sankyo Austria GmbH and Shire Austria GmbH. MW has received support for congress travels and speaker fees from CSL Behring, Boehringer Ingelheim RCV GmbH & Co KG Austria and Mitsubishi Tanabe Pharma. AP, AB, PJ, FE, and KH declare that they have no commercial, financial, or personal conflicts of interest to disclose regarding the work presented here.

## AUTHOR CONTRIBUTIONS

JG: design of the study, data collection, data analysis, and drafting the manuscript. AP: data collection, data analysis, and revision of the manuscript. ES: design of the study and revision of the manuscript. AB: data analysis and revision of the manuscript. PJ: data collection and revision of the manuscript. FE: design of the study and revision of the manuscript. KH: data collection and revision of the manuscript. MW: design of the study, data analysis, and revision of the manuscript.
